# Effectiveness of Physiotherapy for Improving Functionality, Participation, and Quality of Life after a Stroke: Study Protocol for a Randomized Controlled Clinical Trial

**DOI:** 10.3390/jpm14080891

**Published:** 2024-08-22

**Authors:** Concepción Soto-Vidal, Victoria Calvo-Fuente, Ezequiel Hidalgo-Galante, Ester Cerezo-Téllez, Yolanda Pérez-Martín, Soraya Pacheco-da-Costa

**Affiliations:** 1 Neuromusculoskeletal Physical Therapy in Stages of Life Research Group (FINEMEV), Department of Nursing and Physical Therapy, Faculty of Medicine and Health Sciences, Universidad de Alcalá, Autovía A2, km 33.200, Alcalá de Henares, 28805 Madrid, Spain; conchi.soto@uah.es (C.S.-V.); ester.cerezo@uah.es (E.C.-T.); soraya.pacheco@uah.es (S.P.-d.-C.); 2 Physical Medicine and Rehabilitation Service, Ramón y Cajal University Hospital, Ctra. Colmenar Viejo km 9.100, 28034 Madrid, Spain; ezequiel.hidalgo@salud.madrid.org; 3 Humanization in the Intervention of Physiotherapy for the Integral Attention to the People (HIPATIA), Department of Nursing and Physical Therapy, Faculty of Medicine and Health Sciences, Universidad de Alcalá, Autovía A2, km 33.200, Alcalá de Henares, 28805 Madrid, Spain; yolanda.perez@uah.es

**Keywords:** stroke, physical therapy, clinical trial, functional goals, task-oriented, task-specific training

## Abstract

Background: Stroke survivors experience significant alterations in their daily functionality that has a negative impact on their functionality, participation, and quality of life. Person-centered approaches in Physical Therapy interventions that are focused on functional and meaningful goals help to minimize the impact of the alterations. Therefore, the aim of this study is to assess the effectiveness of a Physical Therapy intervention based on a goal-oriented approach with task-specific training for improving functionality, participation, and quality of life for people with Stroke. Methods: A single-blinded randomized controlled clinical trial will be developed. Adults over 50 years old diagnosed with Stroke over 6 months will be included in this study. Participants (n = 62) will be randomly allocated into two groups: The experimental group (n = 31) will receive 30 sessions, three per week during 10 weeks, of Physical Therapy sessions of goal-directed and task-specific training. The control group (n = 31) will follow the same intervention intensity of their usual Physical Therapy treatment. The primary outcome variables quality of life (NewsQol), participation (Ox-PAQ), and gait functionality (FAC) and the secondary outcome variables functional disability (BI), postural control (PASS), dynamic trunk balance (TIS), and functional goals (GAS) will be measured at baseline, after group interventions (10 weeks), and 6 months after the baseline. Statistical analyses will include repeated-measures ANOVA, Student’s *t*-test, or the Mann–Whitney U-test, with a 95% confidence interval and significance level of *p* < 0.05. Conclusion: Person-centered approaches in Physical Therapy interventions may yield better outcomes in functionality, participation, and quality of life for Stroke patients compared to standardized interventions. Trial registration: ClinicalTrials.gov: NCT06165666 (December 2023).

## 1. Introduction

Stroke stands as the foremost cause of physical disability and the second leading cause of mortality among adults in developed nations. This medical condition not only affects the quality of life of individuals but also imposes a significant socio-economic burden on healthcare systems and economies [1,2]. The financial impact is considerable, with the average annual cost per patient in high-income countries estimated to be USD 27,702. Notably, the United States incurs the highest average cost per patient, at USD 59,900, followed by Sweden at USD 52,725 and Spain at USD 41,950 [3]. These figures underscore the critical need for effective prevention, early intervention, and efficient management strategies to reduce both the incidence of Strokes and the financial strain they place on healthcare systems and society at large [1,2,3].

Worldwide, the number of people suffering from Stroke has increased significantly in the recent decades [4]. This increase has been exacerbated by the COVID-19 pandemic, as Stroke is one of the potential complications associated with the virus [5,6]. The prevalence of Stroke notably increases with age, particularly among those over 65 years old. It is estimated that approximately 32.4% of Stroke survivors experience some form of functional dependence six months after the event. This highlights the growing public health challenge and the need for targeted interventions to manage and mitigate the long-term impacts of Stroke [7].

Among the most common long-term effects of Stroke, functional impairments are the leading cause of disability. These impairments can significantly impact balance, leading to gait disturbances and an increased risk of falls. Additionally, they often result in difficulties with handling and performing activities of daily living (ADLs), such as dressing, bathing, and eating [8]. When these alterations continue over time, the negative impact is not only on functionality for ADL performance, but also with the person’s interaction with their environment and participation in social activities [9]. This reduced social engagement can further impact their perceived quality of life [10]. Moreover, the burden of these challenges often extends to family members, who may experience increased stress, social isolation, and decreased life satisfaction [11].

In recent years, a paradigm shift has emerged in the approach to addressing functional dependence among Stroke survivors, guided by the International Classification of Functioning, Disability, and Health (ICF) framework [12,13]. This approach emphasizes the importance of focusing on enhancing functionality and participation rather than solely addressing issues related to body structure and function [14]. This method encourages patients to work towards meaningful goals, which not only aids in their physical recovery but also enhances their overall quality of life by promoting greater independence and social participation [15].

Therefore, it is crucial to emphasize the importance of person-centered approaches in Physical Therapy (PT) interventions to help individuals maximize health, well-being, functionality, quality of life, and participation [16,17]. Person-centered interventions, which focus on functional and meaningful goals, are essential for improving performance. These approaches require the individual’s active involvement in the decision-making process, making them co-responsible for their treatment and subsequent progress [18,19,20]. The interactions between patients and their therapists during the goal-setting process are critical for developing an effective personalized treatment program [18]. This collaborative approach not only enhances the effectiveness of the intervention but also empowers patients, fostering a sense of ownership and motivation in their progress [18,19,20].

The European Stroke Organisation highlights that during motor recovery, patients can optimize their motor, sensory, and cognitive functions by engaging in goal-oriented, repetitive, and progressively challenging task-specific training [7,21,22]. Therefore, PT interventions should also include, among others, the training of functional activities, using task-oriented or task-specific training as an optimal approach strategy for optimizing outcomes [22,23,24]. Including goal-directed approaches and/or task-specific training can enhance PT interventions, leading to improved recovery outcomes for Stroke survivors. This approach has been associated with reduced dependency, lower rates of depression, and increased quality of life and participation [21,22,23,24]. However, more intervention studies with larger sample sizes and detailed methodologies are needed to further validate these findings and refine the implementation strategies [25].

Therefore, the aim of this study is to assess the effectiveness of a PT intervention based on goal-oriented approaches and task-specific training for improving functionality, participation, and quality of life for people with Stroke.

## 2. Materials and Methods

The study protocol follows the Standard Protocol Items: Recommendations for International Trials (SPIRIT) [26].

### 2.1. Study Design

**Figure 1:**
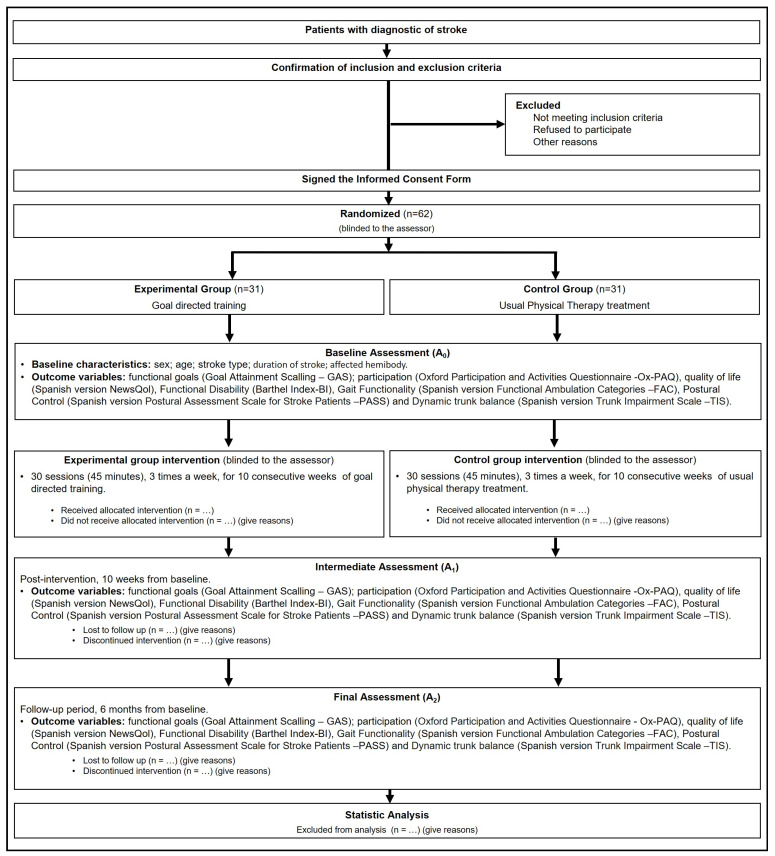
Consort diagram: flow of participants throughout this study.

A single-blinded randomized controlled trial will be conducted according to Consolidated Standards of Reporting Trials (CONSORT) guidelines [27]. Figure 1 shows the flow chart of this study.

### 2.2. Ethics Approval and Consent to Participate

This study was approved by the Drug Research Ethics Committee of the Ramon and Cajal University Hospital with code 220/23, and it was registered in the www.clinicaltrials.gov database under identifier NCT06165666.

This RCT complies with the ethical principles of the Declaration of Helsinki [28] and the Spanish Law on Personal Data Protection and Guarantee of Digital Rights [29]. Information sessions about this study will be held for participants where an information sheet will be provided. Informed and written consent will be obtained from all participants before their inclusion into the trial.

### 2.3. Sample Size Calculation

Sample size was calculated using G*Power software 3.1.9.7© (Germany) [30]. In order to detect differences in quality of life in favor of the EG, a large effect size of d = 0.80, an error α = 0.05, and a power of 90% were assumed. Considering a possible loss to follow-up of 10%, the sample size results in 62 participants.

### 2.4. Study Sample

The subjects will be recruited at the Ramon and Cajal University Hospital Rehabilitation Unit in Madrid (Spain). Those diagnosed with Stroke who attend the unit will be referred to a Physical Therapist, a member of the research team, who will check the inclusion and exclusion criteria, before inviting them to participate in this study.

The inclusion criteria are subjects diagnosed with Stroke between 6 and 24 months before starting this study, over 50 years old. The exclusion criteria are subjects who present moderate or severe cognitive impairment according to the Pfeiffer Questionnaire [31], have other serious diseases with a significant impact on functional capacity, and suffer a previously diagnosed psychiatric pathology or were dependent with regard to basic activities of daily living before suffering the Stroke.

Potential participants will be provided with all the relevant information through the information form and further verbal explanation. They will be told that they will be randomly allocated either in the experimental or control and that each group will be treated with different PT techniques appropriate for their condition, and that the aim of this study is to determine the best results. The patients will not be informed about which group they are assigned. If they decide to participate, they will be asked to sign the informed consent form and, afterwards, 2 Physical Therapists, members of the research team, will carry out the baseline assessment.

### 2.5. Randomization and Blinding

Once the inclusion and exclusion criteria are revised, and after every participant signs the informed consent, participants will be included in this study and codified with a number from 0 to 62.

Randomization will be computer-generated by the statistics program Epidat v.4.1 (La Coruña, Spain) with a 1:1 proportion to the experimental group (EG) or control group (CG). This assignment will be blinded to the assessor.

Only the Physical Therapists who will carry out the interventions will have access to randomization.

### 2.6. Study Flow

After randomization, sociodemographic, clinical, and outcome variables will be collected at baseline assessment (A0). PT interventions will be carried out in each group for 10 weeks. After group interventions, an intermediate assessment (A1) will be performed, and a follow-up assessment (A2) will be carried out 6 months after A0.

### 2.7. Outcome Variables

Outcome variables will be collected at 3 points: at baseline (A0), at the end of the group interventions (A1), and 6 months after baseline (A2). The assessments will be carried out by 2 Physical Therapists, members of the research team, after training and consensus meetings. The assignment will be blinded to the two physical therapist assessors. Sociodemographic variables (Table 1) are age, sex, duration of Stroke, work status, type of Stroke, affected hemibody and comorbidities.

*Primary variables* (Table 2) are as follows:

- Quality of Life: Measured with the Spanish version of Newcastle Stroke-Specific Quality of Life Measure (NewsQoL), developed by Buck et al. [32] to measure the quality of life of people after a Stroke. It consists of 56 items distributed in 11 dimensions. The range of scores varies from 0 to 3 points for each of the items. In each dimension, a result is obtained from 0% to 100%, with a higher score representing a worse perception of quality of life. It is adapted and validated in the Spanish population [33] and it has proven to be sensitive and reliable [34].
- Participation: Measured with the Oxford Participation and Activities Questionnaire (Ox-PAQ), an instrument for measuring participation and activity levels based on the International Classification of Functioning, Disability and Health (ICF) [35]. It consists of 23 items divided into 3 dimensions: routine activities, social commitment, and emotional well-being. Each item is scored on a Likert scale from 0 to 4 points. Higher results mean greater difficulty in participation [35]. It is valid for people who have suffered a Stroke [36], and the Spanish version is up to be done.
- Functionality: Measured with the modified Barthel Index [37], used to assess the functionality of activities of daily living of people with neuromuscular and/or musculoskeletal pathology, especially in those who have suffered Stroke. The total score ranges from 0 to 100, where 0 indicates complete dependence and 100 indicates complete independence. It is cross-culturally adapted and validated in the Spanish population [38].

**Table 2:** Outcome variables.

Outcome Variables	Assessment Period			Measuring Instruments
	Baseline	Post-Treatment	Six Months after Baseline	
Perception of Quality of Life	x	x	x	NewsQoL-S.V.
Participation in the environment	x	x	x	Ox-PAQ
Functional disability	x	x	x	BI
Gait functionality	x	x	x	FAC-S.V.
Postural control	x	x	x	PASS-S.V.
Dynamic trunk balance	x	x	x	TIS-S.V.
Functional goals	x	x	x	GAS

S.V.: Spanish version; NewsQol: Newcastle Stroke-specific Quality of Life Measure; Ox-PAQ: Oxford Participation and Activities Questionnaire; BI: Barthel Index; FAC: Functional Ambulation Categories; PASS: Postural Assessment Scale for Stroke Patients; TIS: Trunk Impairment Scale; GAS: Goal Attainment Scaling.

Secondary variables (Table 2) are as follows:

- Level of gait functionality: Measured with the Spanish version of the Functional Ambulation Categories (FAC). The classification ranges between level 0, which indicates that the subject is unable to walk, and level 5, where the person can walk normally. It was validated in the Spanish population by Viosca et al. [39] and has shown to be sensitive for measuring changes in gait in people who have hemiparesis after suffering a Stroke [40].
- Postural control: Evaluated through the Spanish version of the Postural Assessment Scale for Stroke Patients (PASS) [41], a specific instrument to evaluate postural control and balance. The total score varies from 0 to 36 points, with the interpretation being that the higher the score, the better the person’s balance. Validated in the Spanish population in 2015 [42].
- Dynamic trunk balance: Measured with the Spanish version of the Trunk Impairment Scale (TIS), developed by Verheyden et al., and adapted to the population with Stroke [43]. It consists of two dimensions: dynamic balance in sitting and coordination. The range of scores varies from 0 to 16 points, with higher scores indicating greater trunk control. It is validated for the Spanish population by Cabanas et al. [44].
- Functional goals: Assessed with the Goal Attainment Scaling (GAS), which is a 6-point-scale for measuring significant and realistic functional goals, and demonstrated to be a feasible outcome measure among subacute and chronic Stroke patients [45].

### 2.8. Interventions

The interventions will be performed by four Physical Therapists with more than 15 years of experience in the field of neurological PT, who will be blinded to the assessments. Both groups, the EG and the CG, will receive 30 PT sessions for 40 min, 3 times a week, for 10 weeks.

#### 2.8.1. Experimental Group Intervention

The EG intervention will be based on functional training with specific tasks related to participants’ daily routine, such as personal hygiene (using the toilet, washing face, brushing teeth, using a towel to dry off), dressing/undressing independently (putting on and changing upper and lower body clothing), preparing food (preparing a pot, turning on the stove, placing prepared food on a plate), basic housecleaning (washing dishes, placing dishes on a rack, using a vacuum cleaner, plugging in and off the houseware), walking and shopping in a store near the home (walking to the store, making a purchase, paying for the product, walking back home), and enjoying simple leisure activities [22,23,24,25].

Each session will begin with the analysis of the characteristics of the task (10 min) to determine the abilities that the person presents and to detect the patterns of movement and components (physical, sensitive, sensorial, social, and environmental) that can be trained in order to improve the performance of the task. The training will be performed progressively, prioritizing the key components of each pattern of movements that will be performed actively by the participants, with successive repetitions (25 min). The last part of the session (15 min) will include the training of the task in different situations and settings.

Therefore, each task, aligned with individual functional goals, will be carried out progressively and systematically. Complex activities will be broken down into simpler tasks, with the difficulty gradually increasing as the sessions progress [23].

The environment/setting is also very important because it can influence the task to be performed and the motor learning process. Therefore, in each session, there will be modifications according to the level of motor learning to facilitate progressive adaptation to different situations, from a controlled environment to a natural environment [46].

The Physical Therapist will consistently work to maintain the individual’s attention and active participation, facilitating the achievement of quality functional movements. This will be achieved by using repetitive activities with variations to promote motor relearning [46].

#### 2.8.2. Control Group Intervention

Participants assigned to the CG will continue with their standard PT sessions. These sessions will focus on maintaining joint range of motion, practicing transfers from sitting to standing and from standing to sitting, maintaining posture while seated with or without assistance, and performing coordination exercises. Additionally, the CG will engage in activities designed to enhance balance and stability, ensuring a comprehensive approach to preserving and improving functional mobility.

### 2.9. Statistical Analysis

The Statistical Package for the Social Science software (SPSS^®^ v25) for Mac will be used. An intention-to-treat analysis will be performed. Tests will be carried out with a 95% confidence interval and a statistical significance level of *p* < 0.05. First, descriptive analysis will be performed using means and standard deviations or medians and interquartile ranges otherwise. Normality will be studied through Kolmogorov–Smirnov’s test. For nominal qualitative variables, absolute and relative frequencies will be used. The homogeneity of descriptive and baseline data will be analyzed through Student’s *t*-test for independent samples, Welch’s *t*-test, or the Mann–Whitney U-test. In the case of qualitative variables, Pearson’s χ2 or Fisher’s exact test will be used. Baseline differences will be adjusted through covariate adjustment in regression models. ANCOVA might also be performed to control for baseline differences while comparing the means of the outcome between groups.

To analyze within-group differences between baseline, 6-week, and 6-month assessments, repeated-measures ANOVA or Friedman’s ANOVA will be carried out. To estimate the effect size, partial eta squared will be used. On the other hand, to study the differences between groups, Student’s *t*-test for independent samples, Welch’s *t*-test, or the Mann–Whitney U-test will be carried out. The effect size will be estimated using Cohen’s d or following Grissom’s criteria [47,48,49] in the case of the Mann–Whitney U-test.

Finally, the possible effect of the interaction between descriptive data and independent variables (EG and CG) on the dependent variables will be analyzed through ANCOVA, estimating the effect size using partial eta squared.

Intention-to-treat analysis will be performed in order to reduce, among others, the attrition bias and selection bias that could distort the results. Sensitivity analysis will also be performed in order to address the impact of missing data and ensure the results are as reliable and unbiased as possible.

## 3. Discussion

The aim of this study is to evaluate the effectiveness of a PT intervention that emphasizes functional goal-directed and task-specific training. The primary focus is to assess improvements in functionality, participation, and quality of life for individuals who have had a Stroke. According to the authors’ knowledge, this is the first study to implement a person-centered intervention that integrates goal-directed and task-specific approaches in order to measure the impact on participation and quality of life for Stroke survivors. Therefore, this study intends to bridge the gap in current research by not only assessing traditional functional outcomes, but also focusing on the broader aspects of participation and quality of life. The intervention is designed to be tailored to the individual needs and goals of each participant, reflecting a holistic approach to rehabilitation that goes beyond conventional PT practices. By measuring outcomes related to participation, this study seeks to provide a comprehensive understanding of how these specialized interventions can facilitate reintegration into daily and social activities and enhance overall well-being. The findings of this study could have significant implications for the development of more effective, personalized intervention protocols for Stroke survivors.

Participation and quality of life perception are closely related aspects that are fundamental to a person’s recovery after having a Stroke. Evidence suggests that, within the first three months post Stroke, individuals often express dissatisfaction with their ability to participate in social and daily activities [9]. This dissatisfaction frequently persists over the long term, emphasizing the need for intervention in this area. Despite this, contemporary models of PT interventions usually tend to concentrate primarily on body structure and function impairments and improving gait mobility, motor control, and balance [7,21,24]. Most of these interventions operate under the assumption that enhancements in these areas will naturally lead to a restoration of functionality and the impact on other areas. There is growing recognition that interventions should not be limited to addressing impairments in body structure and function. Instead, they should also emphasize enhancing participation and quality of life [17,18,19]. This holistic approach acknowledges that true recovery encompasses more than just physical rehabilitation; it involves enabling individuals to engage fully in their desired activities and social roles [50]. Therefore, PT interventions should be designed to support not only the physical rehabilitation of Stroke survivors but also their reintegration into the community and social life [51]. By prioritizing participation and quality of life, these interventions can more effectively address the comprehensive needs of Stroke survivors, helping them to regain a sense of autonomy and fulfillment in their everyday lives. This shift in focus is essential for developing a more inclusive and person-centered approach to Stroke rehabilitation [17,18,19].

In contemporary rehabilitation practice, there is a growing trend towards PT interventions that are based on person/family-centered approaches. These approaches emphasize functional goal-oriented and task-specific training, aiming to empower patients and actively involve them in their recovery. Such methodologies not only encourage patients to take an active role in their therapeutic process but also stimulate their motivation, which is crucial for sustained engagement and progress during recovery [15,22,23,24,25]. The literature supports the effectiveness of person-centered goal setting within Stroke interventions, considering that it is feasible and beneficial. These approaches allow the customization of the treatment program to be aligned with the individual needs, preferences, and goals of each person, thus enhancing the relevance and impact of the interventions. Despite the recognized advantages, this practice is not yet widely implemented in clinical settings. The underutilization of person-centered goal setting may be attributed to a variety of factors, including traditional training models, time constraints, and a lack of resources or awareness among healthcare providers [52]. To optimize Stroke intervention outcomes, it would be advisable that more PT practitioners adopt and integrate person-centered strategies. This shift could lead to more tailored and effective interventions that address physical impairments and support patients’ psychological and emotional well-being. By creating a more collaborative therapeutic environment, these approaches can help patients achieve meaningful recovery goals, thereby improving their overall quality of life and satisfaction with the recovery process.

Some PT interventions focus specifically on primary impairments of Stroke, such as spasticity and/or muscle weakness, and it seems that there is not enough evidence on functionality improvement [53]. Therefore, interventions that prioritize the use of active strategies that combine the analysis of movement patterns with training of functional tasks show improvements not only in functionality, but also in muscle strength and/or spasticity [7,21,22,23,24]. The aim of this study, based on the ICF [12,13,14], is addressing secondary impairments that are significant to Stroke survivors, rather than addressing only primary impairments such as spasticity and muscle weakness, although this does not mean that they are not addressed indirectly.

The hypothesis of this study posits that the PT intervention designed and proposed for the EG will be more successful for recovery outcomes, particularly in terms of functionality, quality of life, and participation. To validate this hypothesis, specific outcome variables will be meticulously measured using tailored assessment tools designed for Stroke survivors. This approach ensures that the data collected are both relevant and sensitive to the unique challenges faced by this population [54].

This study will include a significant sample size of 62 individuals aged 50 or older. This demographic is particularly relevant as the incidence of Stroke significantly increases within this age range [4]. Additionally, the study participants will have a post-Stroke evolution time of at least six months. This duration is considered sufficient for individuals to have a clearer understanding and awareness of the changes in their functional abilities, social engagement, and overall quality of life.

The number of sessions, their frequency, and their duration in this study are determined based on established guidelines from the literature and experts’ recommendations [7,55,56,57]. These parameters are carefully chosen to align with best practices in Stroke rehabilitation, ensuring that the intervention is both evidence-based and practical for implementation.

As a novel aspect, the achievement of functional goals is included as an outcome variable. This decision is informed by recommendations from existing research, which underscores the importance of goal attainment in the rehabilitation process [52]. While the measurement of functional goal achievement is recognized as a valuable indicator of rehabilitation success, it has seldom been evaluated prospectively in previous studies. This study aims to fill that gap by rigorously tracking and assessing the extent to which participants meet their individualized functional goals over the course of the intervention and follow-up period. The assessment of functional goals will also contribute valuable data to the field of Stroke rehabilitation, potentially highlighting the importance of goal-oriented approaches in enhancing patient outcomes [20].

This study may face some limitations such as the lack of adherence for the duration of this study and further complications because of the age of the participants.

## 4. Conclusions

This study could provide valuable insights into the effectiveness of person-centered, goal-directed, and task-specific interventions, offering further understanding of how these approaches can be optimized to support Stroke survivors in achieving meaningful recovery.

## Figures and Tables

**Table 1:** Sociodemographic variables.

Variable	Values
Age	Years
Sex	Man/Woman
Stroke evolution time	Months
Work status prior to the Stroke	Employed/Unemployed
Actual work status	Employed/Unemployed
Type of Stroke suffered	Ischemic/Hemorrhagic
Most affected hemibody	Right/Left
Other comorbidities	Yes/No

## Data Availability

The data generated in this study will be included in the results of the published article.
